# *Bartonella henselae* Hepatic Abscesses and Associated Osteomyelitis in a Pediatric Patient

**DOI:** 10.1155/2024/7810497

**Published:** 2024-08-05

**Authors:** Molly Antonson, Lauren Klingemann, Kari Neemann

**Affiliations:** ^1^ College of Medicine University of Nebraska Medical Center, Omaha, NE, USA; ^2^ Division of Pediatric Infectious Disease University of Nebraska Medical Center, Omaha, NE, USA

## Abstract

*Bartonella henselae* is a Gram-negative bacillus transmitted to humans via cat saliva or scratch. Cat scratch disease, the typical clinical manifestation of *B. henselae* infection, presents as localized cutaneous or regional lymphadenopathy. Rare, atypical presentations, generally reflecting bloodborne disseminated disease, can include hepatosplenic, cardiac, ocular, neurologic, or musculoskeletal involvement. Here, we present a case of disseminated *B. henselae* with hepatic abscesses and associated ischial osteomyelitis in an immunocompetent 2-year-old male patient. Although osteomyelitis is a rare manifestation of *B. henselae* infection, it should be included in the differential diagnosis in pediatric patients presenting with fever of unknown origin and musculoskeletal pain, especially in the setting of cat exposure. Hepatic involvement of *B. henselae* infection is associated with significant morbidity; therefore, abdominal imaging is critical in the diagnostic workup. This patient was successfully treated after a 6-week course of azithromycin and rifampin, as evidenced by symptom resolution and improved fluid collections on repeat imaging. While most cases of *B. henselae* resolve without treatment, in severe or disseminated infection such as this case, antibiotics such as azithromycin and rifampin should be considered for treatment.

## 1. Introduction

*Bartonella henselae* is an aerobic, oxidase-negative intracellular Gram-negative bacilli [1]. Cat fleas spread the bacterium among cats, particularly in kittens allowed outdoor roaming, which can then pass it to humans through scratches or bites [2]. Cat scratch disease (CSD), the typical clinical manifestation, presents in 85–90% of children as localized cutaneous or regional lymphadenopathy [3, 4]. It typically follows a self-limiting course, and therapeutic use of antimicrobials is seldom necessary. Children aged 5–9 have the highest rates for both outpatient and inpatient diagnoses. CSD incidence varies regionally with flea abundance, being highest in the warm, humid South and lowest in arid, mountainous areas [2]. The incidence of patients hospitalized with *B. henselae* is estimated to be 0.77–0.86 per 100,000 children less than five years old [1]. Although poorly understood, disseminated disease is thought to be due to bacteria adhering to and invading erythrocytes, facilitating intracellular replication [5]. Atypical presentations generally reflect disseminated disease in immunocompromised patients and can include hepatosplenic, cardiac, ocular, neurologic, or musculoskeletal involvement [3, 4, 6–10]. Bone involvement is especially uncommon, with osteomyelitis reported in 0.27% of patients with *Bartonella henselae* infection [11].

## 2. Case Presentation

**Figure 1:**
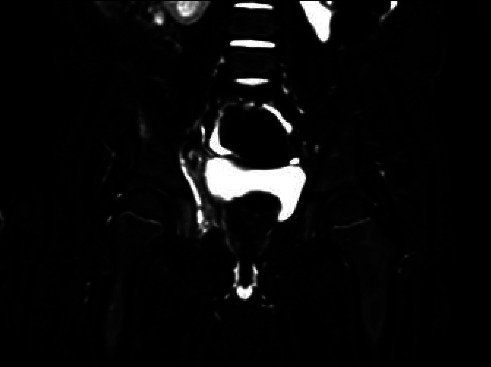
MRI pelvis. Right hemipelvis edema with probable osteomyelitis. Intraosseous abscess within the right ischium with surrounding myositis.

A previously healthy, fully vaccinated two-year-old male was admitted with two and a half weeks of persistent fever and one day of right leg pain and limp. Before admission, the patient underwent evaluations in various medical settings. He received an early diagnosis and was treated with intramuscular penicillin G benzathine for Group A streptococcal pharyngitis based on positive polymerase chain reaction (PCR) testing, despite the absence of a sore throat. Additionally, he was prescribed a one-week course of cefdinir due to persistent fevers without improvement in symptoms. The patient's past medical and family histories were noncontributory. Pertinent social history revealed a mice infestation in the home and a trip to Guatemala six months prior without malaria prophylaxis. The exam was notable for fever (38.6 degrees Celsius), a right-sided limp with ambulation, and hip pain with extension but a full range of motion otherwise. Laboratory findings on admission were notable for leukocytosis (white blood cell count 14.4 10^9^/liter), anemia (10.1 grams/deciliter), elevated inflammatory markers (erythrocyte sedimentation rate 80 millimeters/hour, c-reactive protein 3.2 milligrams/deciliter) and negative blood cultures. Magnetic resonance imaging (MRI) of his right hip revealed an intraosseous abscess within the right ischium with surrounding myositis but no joint involvement (Figure 1).

**Figure 2:**
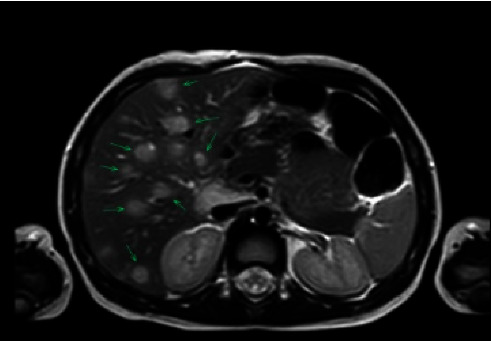
MRI pelvis. Arrows pointing to innumerable small hepatic ring enhancing lesions.

**Figure 3:**
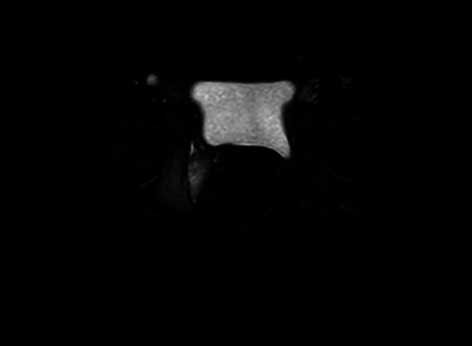
MRI pelvis. Right pelvic osteomyelitis, now with adjacent tissue abscess formation.

Infectious Diseases was consulted, and the patient was started on intravenous cefazolin to target common bacterial pathogens associated with osteomyelitis in this age group (*Staphylococcus aureus*, *Streptococcus pyogenes*, and *Kingella kingae*) [12]. Although the right leg limp resolved, fevers persisted. On the eighth day of hospitalization, MRI short tau inversion recovery (STIR) performed to evaluate for additional sites of infection found innumerable transverse relaxation time (T2) hyperintense lesions throughout the liver in addition to known pelvic osteomyelitis. Dedicated abdominal MRI confirmed innumerable small hepatic ring-enhancing lesions and the formation of abscesses in the pelvis (Figures 2 and 3).

After further investigation and questioning, it was discovered that the patient's family had a pet cat that died four to six weeks prior to hospital admission. They had also recently adopted two new kittens, although the exact timeline on this was unclear. Despite negative pelvic abscess cultures, both serology (immunoglobulin G 1 : 1024; immunoglobulin M 1 : 16) and broad-range PCR (*B. henselae* deoxyribonucleic acid (DNA) detected with 16S ribosomal ribonucleic acid (RNA)) identified *Bartonella henselae* as the pathogen. Brucella serology and *Mycobacterium tuberculosis* complex PCR were both negative. The patient received a 6-week treatment course of azithromycin and rifampin, with resolution of presenting symptoms and improved fluid collections on repeat imaging.

## 3. Discussion

Confirmed cases of *B. henselae* infection are rare secondary to their often-self-limiting nature. Blood and tissue cultures are often negative [11]. Therefore, tests such as serology and PCR can be utilized in the diagnostic evaluation. This case represents an atypical, disseminated *B. henselae* infection with osteomyelitis and hepatosplenic involvement, and is especially unique given the patient's immunocompetent status. *Bartonella* osteomyelitis most often occurs in the spine, followed by the pelvic girdle [13]. The differential diagnosis of the hepatosplenic radiologic findings could indicate numerous unique etiologies. Therefore, positive pathogen detection via PCR, culture, or serological titers is critical in diagnosis. While is debatable whether CSD requires antimicrobial therapy, it can be treated with monotherapy with a macrolide when necessary [11, 14]. Patients with disseminated disease or immunocompromised status, however, typically require dual therapy with tetracycline, fluoroquinolone, or rifamycin [5]. Treatment of disseminated CSD relies on case series as no randomized control trials exist. A treatment duration of 2–8 weeks is typically recommended, with final duration guided by the resolution of imaging findings [3, 15].

## 4. Conclusion

The importance of obtaining an accurate, detailed patient history (including social history such as pets in the house) in every clinical setting is clearly demonstrated by this case. Although osteomyelitis is a rare manifestation of *B. henselae* infection, especially in immunocompetent patients, it should be included in the differential diagnosis in pediatric patients presenting with fevers and musculoskeletal pain, especially in the setting of cat exposure. Hepatic involvement, another atypical manifestation, can cause significant morbidity. Therefore, abdominal imaging should be considered a critical step in the diagnostic workup of fever of unknown origin or when a patient does not respond as expected to the treatment of suspected infection, as in our case. While most cases of *B. henselae* resolve without treatment, in severe or disseminated cases, antibiotics such as azithromycin and rifampin should be considered. Future randomized controlled trials are needed to optimize treatment of deep-seated *B. henselae* infections.

## Data Availability

The data supporting the results of this study include the patient's labs and imaging findings. The source of the data for this study cannot be publicly accessed as it would reveal unique patient identifier information and would be illegal and unethical.

## Abbreviations

CSD: Cat scratch disease
PCR: Polymerase chain reaction
MRI: Magnetic resonance imaging
STIR: Short tau inversion recovery
T2: Transverse relaxation time
DNA: Deoxyribonucleic acid
RNA: Ribonucleic acid.
